# Vitrification of in vitro matured oocytes collected from antral follicles at the time of ovarian tissue cryopreservation

**DOI:** 10.1186/1477-7827-9-150

**Published:** 2011-11-23

**Authors:** Giovanna Fasano, Federica Moffa, Julie Dechène, Yvon Englert, Isabelle Demeestere

**Affiliations:** 1 Research Laboratory on Human Reproduction, Faculty of Medicine, Campus Erasme, Université Libre de Bruxelles, 1070 Brussels, Belgium; 2 Fertility Clinic, Department of Obstetrics and Gynaecology, Erasme Hospital, Université Libre de Bruxelles, 1070 Brussels, Belgium

## Abstract

**Background:**

In the past few years, cryopreservation of ovarian tissue has become an established procedure proposed in many centers around the world and transplantation has successfully resulted in full-term pregnancies and deliveries in human. This prospective study aims to evaluate the feasibility of vitrifying in vitro matured oocytes (IVM) isolated at the time of ovarian tissue cryopreservation to improve the efficiency of fertility preservation programs.

**Methods:**

Oocyte-cumulus complexes were retrieved from freshly collected ovarian cortex by aspirating antral follicular fluid, and were matured in vitro for 24-48 h prior to vitrification. Oocytes were matured in an IVM commercial medium (Copper Surgical, USA) supplemented with 75 mIU/ml FSH and 75 mIU/ml LH and vitrified using a commercial vitrification kit (Irvine Scientific, California) in high security vitrification straws (CryoBioSystem, France). Oocyte collection and IVM rates were evaluated according to the age, the cycle period and the amount of tissue collected.

**Results:**

Immature oocyte retrieval from ovarian tissue was carried out in 57 patients between 8 and 35 years of age, undergoing ovarian tissue cryopreservation. A total of 266 oocytes were isolated, 28 of them were degenerated, 200 were at germinal vesicle stage (GV), 35 were in metaphase I (MI) and 3 displayed a visible polar body (MII). The number of oocytes collected was positively correlated with the amount of tissue cryopreserved (*p *< 0.001) and negatively correlated with the age of the patients (*p *= 0.005). Oocytes were obtained regardless of menstrual cycle period or contraception. A total maturation rate of 31% was achieved, leading to the vitrification of at least one mature oocyte for half of the cohort.

**Conclusions:**

The study showed that a significant number of immature oocytes can be collected from excised ovarian tissue whatever the menstrual cycle phases and the age of the patients, even for prepubertal girls.

## Background

Advances in cancer therapy have improved the long-term survival of patients suffering from malignancies. Thus, the number of young adults wishing to become parents following cancer treatment has significantly increased. However, cancer treatment often involves adverse side effects, including loss of gonadal function and sterility [1,2]. Chemotherapy using high doses of alkylating agents and radiotherapy by ionizing radiation reduces the primordial follicle reserve, which may trigger premature ovarian failure (POF). This represents a major concern for young patients hoping to have children. In this context, all options for maintaining or restoring fertility must be considered [3,4]. Oocytes and embryos can be vitrified after ovarian stimulation or during a natural cycle, but this strategy is not recommended to all patients [5-7]. Furthermore, the number of oocytes or embryos vitrified is often not sufficient for more than one or two transfer attempts.

In the past few years, cryopreservation of ovarian tissue has become an established procedure proposed in many centers around the world in order to store a large amount of primordial follicles prior to gonadotoxic treatment [8-10]. Cryopreservation and transplantation of ovarian tissue have successfully resulted in full-term pregnancies and deliveries in humans [11]. One of the major issues regarding ovarian tissue transplantation is the risk of transmission of cancer cells that may have infiltrated the ovarian tissue before the cryopreservation procedure. In these cases, alternatives include in vitro growth of primordial follicles but unfortunately ovarian tissue culture system is not yet available for human application [12,13]. Furthermore, ovarian tissue cryopreservation preserves the primordial and primary follicles, but not the immature oocytes within the antral follicles that do not survive the procedure. These oocytes could however be recovered and subjected to in vitro maturation (IVM) [14]. As reported by many authors, healthy infants were born following IVM [15,16]. Vitrification is now a widely applied and highly successful approach for cryopreservation in reproductive biology, including for the storage of human oocytes [17-19]. Recently, studies reported almost 100% morphological survival rate after vitrification of in vivo aspirated mature oocytes. The authors also reported in vitro embryo development, implantation and pregnancy rates comparable to those achieved with fresh oocytes [20-22].

The present study assessed the efficiency of IVM and vitrification procedures of the immature oocytes in excised ovarian tissue according to the age of the patients and their menstrual cycles.

## Methods

The procedure was approved by the local ethical committee. It was explained to patients and informed consent was obtained.

### Patients

From November 2008 to December 2010, 57 patients between 8 and 35 years of age (mean age 26), referred to Erasme Hospital for ovarian tissue cryopreservation as part of a fertility preservation program, underwent a combined oocyte vitrification procedure after counseling. Seven patients underwent oophorectomy, while the others had ovarian cortex biopsies. The indications for ovarian tissue cryopreservation were breast cancers (n = 26), hematological diseases (n = 20), gynecological diseases (n = 7), solid malignancies (n = 2) and auto-immune diseases (n = 2). The inclusion criteria for the cryopreservation of ovarian tissue were previously described [8]. Patients treated with chemotherapy before the ovarian tissue cryopreservation procedure were excluded from this study [23].

### Oocyte collection and in vitro maturation

The surgical collection and freezing procedures for the ovarian tissue were described elsewhere [8]. Large biopsies of the ovarian cortex (approximately total of half ovary) were removed by laparoscopy except for patients treated with high-dose alkylating agents and autologous stem-cell transplantation, who underwent a unilateral oophorectomy considering their high risk of premature ovarian failure. Oophorectomy was also required in some ovarian diseases, but only a part of the cortex was designated for cryopreservation in these patients. The ovarian cortex was transported to the IVF laboratory in Leibovitz L-15 medium (Life Technologies, Merelbeke, Belgium) at 4°C within the hour. At arrival, oocyte-cumulus complexes (OCCs) were recovered by aspirating all visible antral follicles using 18-gauge syringe needles. The aspirated follicular fluid was poured directly into a Petri dish and examined for OCCs under a stereomicroscope. The ovarian specimens were transferred into a new dish containing the same medium and carefully dissected in order to obtain small slices of ovarian cortex (0.5-1 cm diameter, 1-2 mm thickness). After dissection of the ovarian tissue, the discarded material was filtered through a cell strainer (Falcon, Cell Strainer 352350, 70 μm Nylon) in a Petri dish (Falcon, Petri dishes 3004, 60 × 15 mm) containing 3-5 ml of IVM Washing Medium (Sage, IVM Kit media) at 37°C on a warm stage or plate to prevent the OCCs from drying in the strainer. After filtering, the collected material was rinsed with pre-warmed IVM Washing Medium and transferred into a Petri dish to search a second time for OCCs under a stereomicroscope. All retrieved OCCs were scored and classified according to the oocyte nuclear stage as germinal vesicle (GV), germinal vesicle breakdown (metaphase I-MI) or metaphase II when a first polar body is visible in the perivitelline space (MII). OCCs with unvisualized oocyte nuclear status due to the compact cumulus cells surrounded it were considered as GV. OCCs were washed at least three times in pre-warmed IVM Washing Medium and transferred into an Organ Tissue Culture Dish (Nunc, 176742, 4 wells dishes) containing 0.5 ml IVM Maturation Medium (Sage, IVM Kit media) supplemented with 75 mIU/ml FSH and 75 mIU/ml LH and incubated at 37°C in a 5% CO_2 _humidified atmosphere. The IVM Maturation Medium was prepared for equilibration at least two hours before the immature oocyte retrieval. The immature OCCs were cultured in the IVM Maturation Medium for 24 to 48 hours.

### Vitrification

Twenty-four hours after IVM, all the OCCs were denuded using a 130 micron finely drawn pipette following one minute of exposure to 80 IU/ml hyaluronidase solution (Sigma Aldrich SrL, UK.). The mature oocytes (MII) were then subjected to vitrification following a standard protocol (Irvine, Vitrification Kit media) using aseptic devices (CryoBiosystem, VHS Kit). The remaining immature oocytes (GV and MI) were kept in IVM Maturation Medium for an additional 24 hours.

Forty-eight hours after IVM, the remaining oocytes that reached MII were vitrified.

### Statistical analysis

Statistical analyses were performed using the Chi-squared, t-Test and Non parametric Mann-Whitney Test as appropriate. Linear correlations between two variables were analyzed by calculation of the r-values (Pearson's moment-correlation coefficient); the significance (two-tailed probability values) of r coefficients were calculated on the basis of the correlation values. Values of *p *< 0.05 indicated statistical significance.

## Results

From the cohort of 57 patients, 19% were using contraception treatment (mean age 23.1, range 17-33), 33% were in the follicular phase of a spontaneous menstrual cycle (mean age 26.3, range 14-35), 28% were in the luteal phase (mean age 27.9, range 22-34), 7% were prepubertal (mean age 9.2, range 8-13), 9% were in post-partum amenorrhea (mean age 31, range 23-35) and for 2 patients, information was unavailable (Table 1). A total of 266 oocytes were retrieved, 28 of them were degenerated (10.5%). In the 238 healthy oocytes, 200 were at germinal vesicle stage (GV), 35 were in metaphase I (MI) and 3 displayed a visible polar body (MII).

**Table 1:** Outcome of immature oocyte retrieved from ovarian cortex according to the menstrual cycle characteristic

Characteristic of the patient's menstrual cycle	Patients n	Mean age ± SEM	Mean fragments of ovarian tissue (range)	Oocytes retrieved n (range)	Mean oocytes retrieved /fragment	Mean oocytes retrieved /patients	Stage at collection (%)			IVM rate
							GV	MI	MII	
OC	11	23.1 ± 1.3	20.1 (10-29)	38 (0-9)	0.17 ± 0.07^a^	3.4 ± 1.06^a^	71%	29%	0%	42.1%
Natural cycle FP	19	26.3 ± 1.5	21.6 (7-32)	69 (0-15)	0.17 ± 0.06^a^	3.6 ± 1.09^a^	80%	19%	1%	27.9%
Natural cycle LP	16	27.9 ± 1.1	18.1 (12-26)	44 (0-13)	0.15 ± 0.05^a^	2.8 ± 0.83^a^	84%	14%	2%	39.5%
Post-partum	5	31 ± 2.2	26.3 (16-36)	33 (1-12)	0.23 ± 0.12^a^	6.6 ± 1.86	91%	6%	3%	28.1%
Unknown	2	29.5 ± 0.5	20-32	8 (0-8)	0.15 ± 0.26	4 ± 4	100%	0%	0%	12.5%
Prepubertal	4	9.2 ± 1.4	31.7 (17-40)	46 (2-22)	0.36 ± 0.28^b^	11.5 ± 4.27^b^	93%	7%	0%	23.9%
Total	57	26 ± 0.9	21.8 (7-40)	238 (0-22)	0.19	4	84%	14.7%	1.3%	31%

GV = germinal vesicle; MI = metaphase I; MII = metaphase II; OC = oral contraception; FP = follicular phase; LP = luteal phase; IVM = in vitro maturation; n = number;

a vs b within the same column, *p *< 0.05

In 15/57 patients, no oocytes were found (26.3%). The number of oocytes collected was positively correlated with the amount of tissue cryopreserved (*p *< 0.001) and negatively correlated with the age of the patients (*p *= 0.005). As shown in Table 1 immature oocytes were retrieved regardless of the menstrual cycle phases. The mean number of oocytes retrieved was however higher in prepubertal compared to post-pubertal patients (Table 1). For post-pubertal patients, no difference in the number of oocyte collected was observed between patients less or over 30 year-old (Table 2).

**Table 2:** Outcome of immature oocyte retrieved from ovarian tissue of post-pubertal patients according to the age

Age	Patients n	Mean age ± SEM	Mean fragments of ovarian tissue (range)	Oocytes retrieved n (range)	Mean oocytes retrieved /fragment	Mean oocytes retrieved /patients	Stage at collection (%)			IVM rate
							GV	MI	MII	
< 30 years	30	23.6 ± 0.71	21 (7-32)	99 (0-10)	0.16 ± 0.02	3.3 ± 0.53	83.8%	15.1%	1%	31.3%
≥ 30 years	23	32 ± 0.40	20.9 (11-36)	93 (0-15)	0.18 ± 0.04	4 ± 1.04	79.6%	18.3%	2.1%	30.3%

GV = germinal vesicle; MI = metaphase I; MII = metaphase II; OC = oral contraception; FP = follicular phase; LP = luteal phase; IVM = in vitro maturation; n = number

The total IVM rate was 31% (20% after 24 h IVM and 11% after 48 h IVM). For patients with natural cycles, the IVM rate was similar whatever the phase of the menstrual cycle. No significant difference was observed in the maturation rate between patients using contraception or those in a natural cycle as well as between pre- and post-pubertal patients. For 3 patients, in vitro matured oocytes (4, 1 and 5 respectively) were fertilized by ICSI and the embryos obtained (1, 1 and 3 respectively) were vitrified.

In more than half of the patients (54%), at least one mature oocyte was vitrified after 24 or 48 hours. In 3/4 prepubertal patients, MII were vitrified, suggesting that the procedure is suitable and more efficient for prepubertal patients where antral follicles are present.

## Discussion

The cryopreservation of ovarian tissue allows the preservation of a large number of primary follicles before gonadotoxic treatment. However, growing immature oocytes from antral follicles are lost during the procedure. Vitrification of in vitro matured oocytes collected after punction of these antral follicles in the excised ovarian tissue before cryopreservation has been proposed as an additional technique to preserve fertility. This procedure may increase fertility restoration potential and may also be an important alternative whereby neoplasic cells can potentially infiltrate ovarian tissue, leading to a risk of disease recurrence after transplantation, as in some hematological diseases or in advanced breast cancer [24,25].

This study shows that isolated immature oocytes from antral follicles can be retrieved from the ovarian tissue biopsy, consequently in vitro matured and vitrified during any phase of the menstrual cycle and whether or not the patient is using oral contraception. The number of oocytes collected is correlated with the number of cryopreserved fragments and the age of the patients. However, the IVM rate is similar whatever the phase of the menstrual cycles or the age of patient. These results suggest that the procedure can be proposed to any patient undergoing ovarian tissue cryopreservation, with the exception of those who have already begun chemotherapy.

The procedure described above, allows the vitrification of matured oocytes in approximately half of the patients although the total IVM rate was lower than the one previously described using oocytes directly retrieved in vivo. In a recent study, a mean oocyte maturation rate of around 70% was achieved following direct oocyte retrieval after hCG injection during a natural cycle [26]. The recent success of in vitro oocyte maturation strategies has been attributed to the improvement of the culture media composition [27-29]. In immature oocytes retrieved from ovarian tissue, the delay in relation to transport and oocyte collection is a major issue and may decrease the efficiency of IVM. Revel et al. [30] first described oocyte collection during the cryopreservation of ovarian tissue in 9 patients, amongst whom 3 attempted IVM. In these patients, 5 of 8 MI oocytes were matured in vitro. Other studies including small number of patients or case reports have been described, confirming the feasibility of the procedure [31-33]. In our cohort, the IVM rate was highly variable from one patient to other ranging from 0 to 100%, with an average of 31%. This result is consistent with the only previous study using a large cohort of 19 patients under 20 years of age, showing that 34% of immature oocytes collected from ovarian tissue before cryopreservation are competent to resume meiosis in vitro [34].

Healthy infants have been born following IVM [35-37], and oocyte cryopreservation by vitrification seems a promising technique that appears to be more effective than the conventional slow-freezing method for mature oocytes vitrified shortly after collection [38-40]. In this context, vitrification results in high survival rates of 89-100% and many successful live births worldwide [41-43]. Given these recent successes, although the efficiency of this combined procedure has to be determined by testing the potential for the implantation of embryos derived from these in vitro matured and vitrified oocytes, it is reasonable to suggest this innovative and non-invasive alternative, under institutional review board supervision. Furthermore, if a male partner is present, the in vitro matured oocytes may be fertilized using the in vitro fertilization (IVF) technique, and the resulting embryos may be cryopreserved. For 3 patients, in vitro matured oocytes were fertilized and the embryos obtained were vitrified. Theses cases are reassuring regarding the oocyte quality as it suggests that these oocytes are competent to be fertilized.

## Conclusion

This study shows that the combination of ovarian tissue cryopreservation and immature oocyte retrieval is feasible whatever the phase of menstrual cycle, the use of oral contraception or the age of the patient. Approximately half of these patients should benefit from this combined procedure, and improvement of the IVM rate may better increase the efficiency of the procedure in the future.

## Competing interests

The authors declare that they have no competing interests.

## Authors' contributions

GF performed the study and wrote the manuscript. FM enrolled the patients, participated in critical discussion and revised the manuscript. JD performed the technical assistance for laboratory procedure and participated in the data collection and analysis. YE is the director of the project, participated in the study design and in critical discussion and revised the manuscript. ID is responsible for the fertility preservation project, participated in the study design and in critical discussion and revised the manuscript. All authors read and approved the final manuscript.
